# Susceptibility‐Guided Versus Empirical First‐Line Therapy of *Helicobacter pylori* Infection in Adults: A Systematic Review and Meta‐Analysis

**DOI:** 10.1111/hel.70125

**Published:** 2026-04-14

**Authors:** Manuel Coelho Rocha, Tiago Gaspar, Carlos Bernardes, Pedro Pimentel Nunes

**Affiliations:** ^1^ Unilabs Lisbon Portugal; ^2^ NOVA Medical School Universidade NOVA de Lisboa Lisboa Portugal; ^3^ Department of Gastroenterology Unidade Local de Saúde de São José Lisboa Portugal; ^4^ Department of Surgery and Physiology, Faculty of Medicine University of Porto (FMUP) Porto Portugal; ^5^ RISE@CI‐IPO (Health Research Network) IPO‐Porto Porto Portugal

**Keywords:** antimicrobial resistance, bismuth quadruple therapy, culture, first‐line, *Helicobacter pylori*, meta‐analysis, PCR

## Abstract

**Background:**

Antimicrobial resistance undermines empirical first‐line regimens for *Helicobacter pylori* . We compared susceptibility‐guided therapy (SGT) versus empirical therapy in adults receiving first‐line treatment, focusing on intention‐to‐treat (ITT) eradication.

**Materials and Methods:**

We searched MEDLINE (PubMed), Web of Science, and Scopus from inception to 30 September 2025 for randomized controlled trials (RCTs) and non‐randomized comparative studies (NRS) in adults with confirmed *H. pylori* . Interventions included phenotypic (culture/E‐test) or genotypic (PCR) SGT applied to biopsy, stool, or gastric juice specimens; comparators were empirical regimens including bismuth quadruple therapy (BQT) and non‐BQT options. The primary outcome was ITT eradication. We pooled risk ratios (RR) with DerSimonian–Laird random‐effects, reported I^2^/τ^2^, and derived 95% prediction intervals (PI). Subgroups were prespecified by comparator family (BQT vs. other) and specimen/method. Multi‐arm studies combined empirical arms within family or split across distinct families.

**Results:**

Forty‐two studies met inclusion criteria; all contributed ITT data. RCTs (k = 33) favored SGT over empirical therapy (pooled RR 1.09, 95% CI 1.05–1.13; I^2^ 73%; 95% PI 0.92–1.30). NRS (k = 12) were directionally consistent (pooled RR 1.15, 95% CI 1.10–1.22; I^2^ 75%; 95% PI 0.99–1.35). In RCTs, effects were neutral‐to‐modest vs. BQT (RR 1.03, 95% CI 0.97–1.10) and clearer vs. other empirical regimens (RR 1.12, 95% CI 1.06–1.18).

**Conclusions:**

In adult first‐line therapy, SGT achieves at least non‐inferior—and often superior—eradication versus empirical regimens; the incremental benefit is attenuated where BQT is standard. These findings support selective, and increasingly justified, integration of susceptibility testing in settings with clinically relevant resistance.

## 1 Introduction

*Helicobacter pylori* ( *H. pylori* ) causes chronic gastritis and underlies peptic ulcer disease, gastric mucosa‐associated lymphoid tissue lymphoma, and gastric cancer. Eradication reduces incident cancer and is now recommended once infection is confirmed. The global burden remains substantial, with marked geographic variation and ongoing transmission early in life [1]. Rising antibiotic resistance, particularly to clarithromycin and fluoroquinolones, has eroded the performance of several legacy regimens. A large WHO‐region meta‐analysis reported primary and secondary resistance rates ≥ 15% for clarithromycin, metronidazole, and levofloxacin in most regions, and showed a strong association between clarithromycin resistance and failure of clarithromycin‐containing therapy [2]. Treatment failures themselves can further amplify resistance and increase minimum inhibitory concentrations (MICs), highlighting the importance of getting first‐line therapy right [3].

Contemporary guidelines therefore emphasize two complementary strategies at the start of care: (i) use of high‐performing empirical regimens such as bismuth quadruple therapy (BQT) where individual susceptibility is unavailable, and (ii) adoption of susceptibility‐guided therapy (SGT) – phenotypic (culture with E‐test or agar dilution) or genotypic (polymerase chain reaction (PCR) or sequencing) – when feasible, within an antimicrobial‐stewardship framework. Molecular assays detecting canonical determinants (e.g., 23S rRNA mutations for macrolides and gyrA for fluoroquinolones) are increasingly accessible and can be applied to biopsy, stool, or gastric juice matrices, whereas culture‐based testing provides MICs across a broader antibiotic panel [4]. Alongside these practice shifts, reviews have noted that non‐bismuth quadruple and vonoprazan‐based triple regimens can achieve high empirical efficacy in some settings, though concerns remain about multi‐antibiotic exposure and resistance selection when therapy is not tailored [5].

Prior evidence syntheses have often mixed first‐line and rescue indications or combined adult and pediatric cohorts, complicating decision‐making at the point of initial therapy. Some have argued that SGT does not clearly outperform updated quadruple regimens when those regimens are used empirically, whereas others support broader testing in specialized programs [6]. In parallel, consensus statements now recognize *H. pylori* gastritis as an infectious disease and advocate either effective empiric therapy (typically BQT) when susceptibility is unknown, or susceptibility testing where available to optimize antibiotic choice.

Therefore, we conducted a systematic review and meta‐analysis restricted to adults receiving first‐line therapy, separating randomized controlled trials (RCTs) from non‐randomized studies (NRS). We compared SGT with empirical regimens, prespecified BQT versus non‐BQT comparators, and stratified by testing matrix/method (culture/E‐test vs. PCR), aiming to provide directly actionable estimates for initial treatment decisions in current resistance ecologies.

## 2 Materials & Methods

### 2.1 Protocol and Reporting

This review followed the PRISMA 2020 guideline [7] and was pre‐registered on PROSPERO (CRD420251138257). The protocol prespecified eligibility criteria, outcome definitions, multi‐arm handling, and the statistical approach, including subgroup and sensitivity analyses.

### 2.2 Eligibility Criteria

We targeted the clinical decision at initial therapy. Eligible studies enrolled adults (≥ 18 years) with confirmed *Helicobacter pylori* infection who were treatment‐naïve for eradication and were randomized or assigned to susceptibility‐guided versus empirical first‐line regimens. The guided approach could be phenotypic (culture with E‐test or agar dilution informing minimum inhibitory concentration‐based selection) or genotypic (PCR or sequencing) applied to gastric biopsy, stool, or gastric juice (string‐test). Genotypic susceptibility testing targeted macrolide (23S rRNA) and fluoroquinolone (gyrA) resistance determinants, and other resistance markers when assessed. Empirical comparators comprised contemporary regimens, including BQT and non‐BQT backbones in current clinical use.

The primary outcome was intention‐to‐treat (ITT) eradication, defined as the proportion of all randomized/allocated participants with confirmed eradication at test‐of‐cure using validated methods (e.g., 13C‐urea breath test, stool antigen, or invasive tests), typically 4–12 weeks after therapy. Per‐protocol (PP) outcomes were extracted when reported but were not the primary summary measure.

We excluded pediatric‐only populations, rescue/salvage regimens, single‐arm or non‐comparative designs, meeting abstracts without full reports, and studies lacking extractable ITT counts. In multi‐arm trials, empirical arms within a single therapeutic comparator family were combined into one comparator to avoid unit‐of‐analysis errors; when distinct families co‐existed (e.g., BQT and non‐BQT), we treated each contrast as an independent comparison under a predefined hierarchy. Further stratification of non‐bismuth regimens (e.g., clarithromycin‐based triple therapy, concomitant therapy, and vonoprazan‐based regimens) was not performed due to inconsistent reporting and limited sample sizes within individual regimen categories.

### 2.3 Information Sources and Search Strategy

We searched MEDLINE (PubMed), Web of Science, and Scopus from inception through September 2025, with no language restrictions and filters for humans and adults. Strategies combined controlled vocabulary and structured title/abstract terms for “ *Helicobacter pylori* ”, “antimicrobial resistance/susceptibility”, and testing modalities (“culture,” “E‐test,” “PCR,” “23S,” “gyrA,” “rdxA,” “pbp1A,” “rpoB,” “16S”), together with constructs for guided versus empirical therapy. We also screened reference lists of included reports. Full database‐specific search strings and date limits are provided in Appendix S1.

### 2.4 Study Selection and Data Extraction

Two reviewers (MCR, TG) independently screened titles and abstracts and then assessed full‐text eligibility using Rayyan [8], with disagreements resolved by a third reviewer (CB). Reasons for full‐text exclusion were recorded in predefined categories. We extracted study design, setting/geography, specimen and susceptibility‐testing approach (phenotypic culture/E‐test vs. genotypic PCR/sequencing, including resistance targets when reported), empirical comparator family (BQT vs. non‐BQT), and eradication outcomes using both intention‐to‐treat (ITT) and per‐protocol (PP) denominators. In addition to the characteristics presented in Table 1, we collected details on regimen components and duration, susceptibility targets, and test‐of‐cure method and timing; these data informed eligibility assessment and subgrouping but are not fully tabulated to preserve readability. For zero‐event cells in a 2 × 2 table, we applied a prespecified continuity correction of 0.5 to all cells. Multi‐arm trials were handled according to the prespecified hierarchy described above.

**TABLE 1:** Characteristics of included studies (adults, first‐line) and ITT eradication outcomes.

Author	Year	Country	Design	Comparator	Specimen	Method	Susceptibility‐guided ITT eradication rate	Empirical ITT eradication rate
Toracchio et al. (2000) [9]	2000	Italy	RCT	Non‐BQT	biopsy	Culture/E‐test	48/53 (90.6)	42/56 (75.0)
Neri et al. (2003) [10]	2003	Italy	RCT	Non‐BQT	biopsy	Culture/E‐test	88/116 (75.9)	78/116 (67.2)
Marzio et al. (2006) [11]	2006	Italy	RCT	Non‐BQT	biopsy	Culture/E‐test	39/41 (95.1)	36/39 (92.3)
Furuta et al. (2007) [12]	2007	Japan	RCT	Non‐BQT	biopsy	PCR	144/150 (96.0)	105/150 (70.0)
Kawai et al. (2008) [13]	2008	Japan	RCT	Non‐BQT	stool	PCR	33/35 (94.3)	25/35 (71.4)
Cosme et al. (2013) [14]	2012	Spain	NRS	Non‐BQT	biopsy	Culture/E‐test	113/134 (84.3)	57/113 (50.4)
Molina‐Infante et al. (2012) [15]	2012	Spain	RCT	Non‐BQT	biopsy	Culture/E‐test	29/42 (69.0)	41/45 (91.1)
Lee et al. (2013) [16]	2013	Korea	RCT	Non‐BQT	biopsy	PCR	176/218 (80.7)	433/616 (70.3)
Martos et al. (2014) [17]	2014	Spain	RCT	Non‐BQT	gastric juice	Culture/E‐test	47/50 (94.0)	36/54 (66.7)
Park et al. (2014) [18]	2014	Korea	RCT	Non‐BQT	biopsy	Culture/E‐test	54/57 (94.7)	41/57 (71.9)
Cosme et al. (2016) [19]	2015	Spain	NRS	Non‐BQT	biopsy	Culture/E‐test	171/182 (94.0)	103/118 (87.3)
Dong et al. (2015) [20]	2015	China	RCT	BQT	biopsy	Culture/E‐test	41/45 (91.1)	33/45 (73.3)
Zhou et al. (2016) [21]	2015	China	RCT	Mixed (BQT + Non‐BQT)	biopsy	Culture/E‐test	282/318 (88.7)	545/700 (77.9)
Tanabe et al. (2018) [22]	2018	Japan	NRS	Non‐BQT	biopsy	Culture/E‐test	198/212 (93.4)	951/1143 (83.2)
Byambajav et al. (2019) [23]	2019	Mongolia	NRS	Mixed (BQT + Non‐BQT)	biopsy	Culture/E‐test	41/46 (89.1)	204/270 (75.6)
Chen et al. (2019) [24]	2019	China	RCT	BQT	biopsy	Culture/E‐test	262/286 (91.6)	82/96 (85.4)
Choi et al. (2019) [25]	2019	Korea	RCT	BQT	biopsy	PCR	48/50 (96.0)	98/104 (94.2)
Delchier et al. (2019) [26]	2019	France	RCT	Non‐BQT	biopsy	PCR	177/207 (85.5)	152/208 (73.1)
Lee et al. (2019) [27]	2019	Korea	NRS	Non‐BQT	biopsy	Culture/E‐test	69/74 (93.2)	97/117 (82.9)
Ong et al. (2019) [28]	2019	Korea	RCT	Non‐BQT	biopsy	PCR	164/201 (81.6)	169/196 (86.2)
Shinmura et al. (2019) [29]	2019	Japan	NRS	Non‐BQT	biopsy	Culture/E‐test	341/379 (90.0)	460/541 (85.0)
Criado et al. (2020) [30]	2020	Spain	RCT	BQT	biopsy	Culture/E‐test	48/53 (90.6)	51/55 (92.7)
Kim et al. (2020) [31]	2020	Korea	RCT	Non‐BQT	biopsy	PCR	32/36 (88.9)	27/36 (75.0)
Pan et al. (2020) [32]	2020	China	RCT	BQT	biopsy	Culture/E‐test	238/310 (76.8)	100/157 (63.7)
Cha et al. (2021) [33]	2021	Korea	RCT	BQT	biopsy	PCR	118/178 (66.3)	142/182 (78.0)
Cho et al. (2022) [34]	2021	Korea	RCT	BQT	biopsy	PCR	114/141 (80.9)	121/141 (85.8)
Choi et al. (2021) [35]	2021	Korea	RCT	Non‐BQT	biopsy	PCR	91/110 (82.7)	88/107 (82.2)
Hsieh et al. (2021) [36]	2021	Taiwan	RCT	Non‐BQT	gastric juice	PCR	81/91 (89.0)	69/91 (75.8)
Kang et al. (2021) [37]	2021	Korea	NRS	Non‐BQT	biopsy	Culture/E‐test	88/103 (85.4)	90/154 (58.4)
Perkovic et al. (2021) [38]	2021	Croatia	RCT	Non‐BQT	biopsy	Culture/E‐test	37/40 (92.5)	28/40 (70.0)
Cummings et al. (2022) [39]	2022	USA	NRS	Non‐BQT	biopsy	PCR	85/101 (84.2)	14/26 (53.8)
Kim et al. (2022) [40]	2022	Korea	RCT	Non‐BQT	biopsy	PCR	124/145 (85.5)	120/145 (82.8)
Li et al. (2022) [41]	2022	China	RCT	BQT	biopsy	Culture/E‐test	108/134 (80.6)	57/67 (85.1)
Choi et al. (2023) [42]	2023	Korea	NRS	Non‐BQT	biopsy	PCR	91/105 (86.7)	85/105 (81.0)
Han et al. (2023) [43]	2023	China	NRS	BQT	gastric juice	PCR	31/32 (96.9)	218/261 (83.5)
Jiang et al. (2023) [44]	2023	China	RCT	BQT	biopsy	PCR	77/85 (90.6)	66/85 (77.6)
Amiot et al. (2024) [45]	2024	France	RCT	Non‐BQT	biopsy	PCR	119/120 (99.2)	116/121 (95.9)
Kim et al. (2024) [46]	2024	Korea	RCT	Non‐BQT	biopsy	PCR	235/287 (81.9)	201/306 (65.7)
Lee et al. (2024) [47]	2024	Korea	RCT	Non‐BQT	biopsy	Culture/E‐test	197/234 (84.2)	65/78 (83.3)
Cho et al. (2025) [48]	2025	Korea	RCT	Non‐BQT	biopsy	PCR	138/150 (92.0)	125/147 (85.0)
Yu et al. (2025) [49]	2025	China	RCT	BQT	stool	PCR	174/199 (87.4)	164/200 (82.0)
Zhou et al. (2025) [50]	2025	China	NRS	BQT	biopsy	PCR	118/125 (94.4)	366/425 (86.1)

Abbreviations: BQT, bismuth quadruple therapy; ITT, intention‐to‐treat; NRS, non‐randomized study; PCR, polymerase chain reaction; RCT, randomized controlled trial.

### 2.5 Risk of Bias and Certainty

Randomized controlled trials were appraised with RoB 2 [51], and non‐randomized comparative studies with ROBINS‐I^10^ [52]. Certainty in the evidence for the primary outcome was judged using GRADE [53] across risk of bias, inconsistency (including between‐study heterogeneity), indirectness (e.g., matrix/method), imprecision (95% confidence interval width and optimal information size), and publication bias. Risk of bias assessments are presented in Table S2, and GRADE judgments in Table S1.

### 2.6 Effect Measures and Statistical Synthesis

The primary effect measure was the risk ratio (RR) for ITT eradication. We pooled log (RR) using DerSimonian–Laird (DL) random‐effects [54] with inverse‐variance weighting, summarized between‐study heterogeneity by I^2^ and τ^2^, and reported 95% prediction intervals (PI) [55] on the log scale to convey the expected range of true effects across comparable settings. The primary synthesis included RCTs; NRS were pooled separately as supportive evidence. Pre‐specified subgroups included empirical comparator (BQT vs. other) and susceptibility testing domain (specimen and method). Sensitivity analyses examined restriction to BQT comparators, restriction by specimen/method (e.g., biopsy‐PCR only), and exclusion of zero‐cell comparisons requiring continuity correction. Small‐study effects were explored using funnel plots and, where ≥ 10 studies were available, formally assessed using Egger's regression test [56].

Because the outcome was eradication, an RR > 1.00 favors susceptibility‐guided therapy. I^2^ quantifies the proportion of variability due to between‐study heterogeneity, τ^2^ is the between‐study variance, and the 95% prediction interval indicates the range in which the true effect of a new, similar study would be expected to lie.

### 2.7 Software and Reproducibility

Analyses were conducted in R (R Foundation for Statistical Computing) using the meta and metafor packages. Title/abstract and full‐text screening were performed in Rayyan (Qatar Computing Research Institute). Code and session details are summarized in Appendix S2.

## 3 Results

**FIGURE 1:**
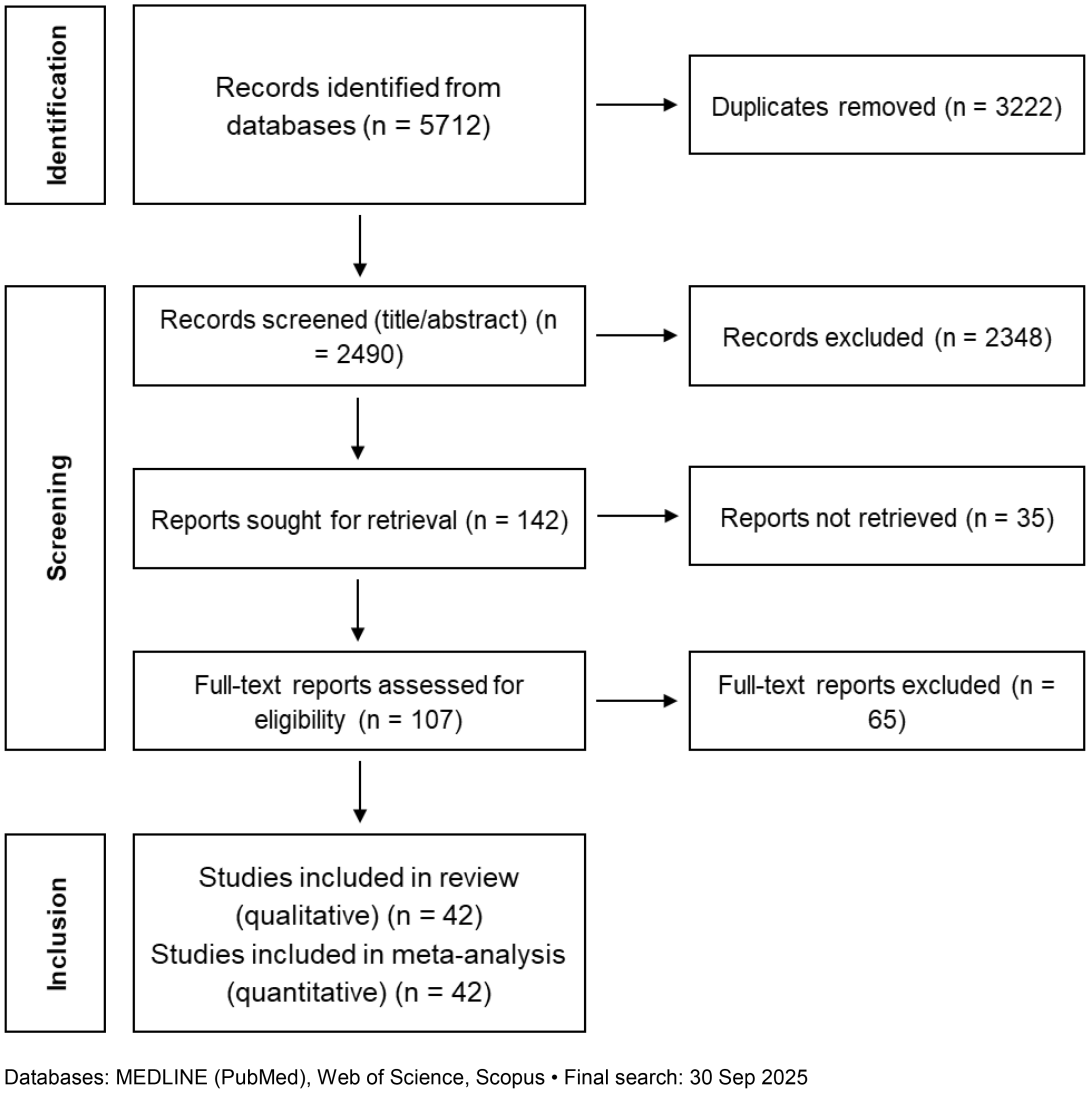
PRISMA flow diagram of study selection.

Database searches yielded 5712 records; after removing 3222 duplicates, 2490 titles/abstracts were screened and 2348 were excluded. We sought 142 full texts and could not retrieve 35; 107 reports were assessed for eligibility. In total, 42 studies met inclusion criteria for the qualitative synthesis, and all 42 contributed ITT data to the quantitative meta‐analysis (Figure 1) [9, 10, 11, 12, 13, 14, 15, 16, 17, 18, 19, 20, 21, 22, 23, 24, 25, 26, 27, 28, 29, 30, 31, 32, 33, 34, 35, 36, 37, 38, 39, 40, 41, 42, 43, 44, 45, 46, 47, 48, 49, 50].

Included studies spanned multiple regions and care settings. Susceptibility guidance was implemented phenotypically (culture/E‐test) or genotypically (PCR), applied to gastric biopsy, stool, or gastric juice (string‐test) specimens. Empirical comparators were most often BQT, alongside contemporary non‐BQT regimens. Study‐level design features, comparator family, specimen/method, and ITT eradication proportions are summarized in Table 1.

**FIGURE 2:**
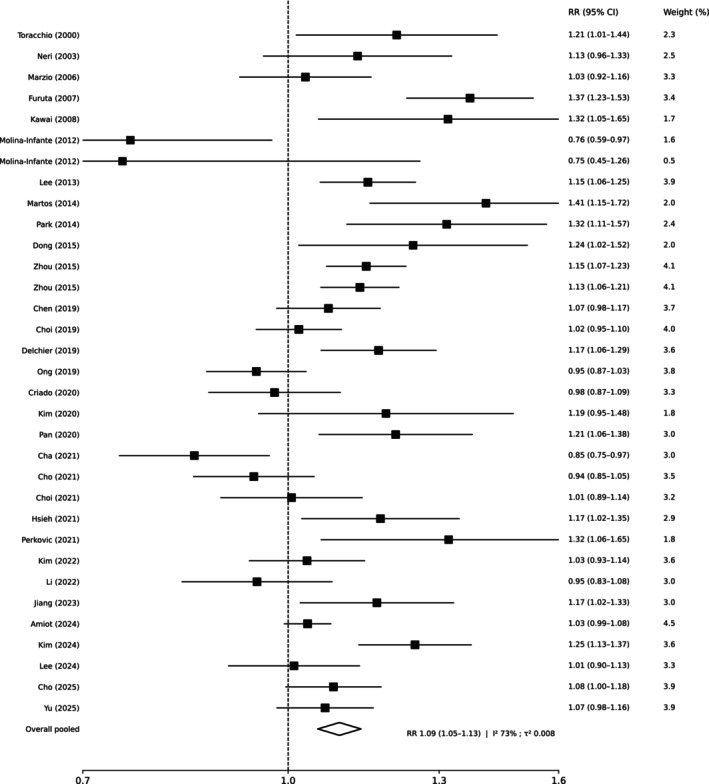
Forest plot of randomized controlled trials comparing susceptibility‐guided therapy with empirical first‐line regimens in adults, showing intention‐to‐treat eradication. Squares represent study‐specific risk ratios (RR) with 95% confidence intervals (CI), with size proportional to study weight; the diamond denotes the pooled random‐effects estimate. The vertical dashed line indicates no difference (RR = 1.0). In trials evaluating more than one empirical regimen family, family‐specific contrasts are displayed as separate lines in accordance with the prespecified multi‐arm handling strategy.

**FIGURE 3:**
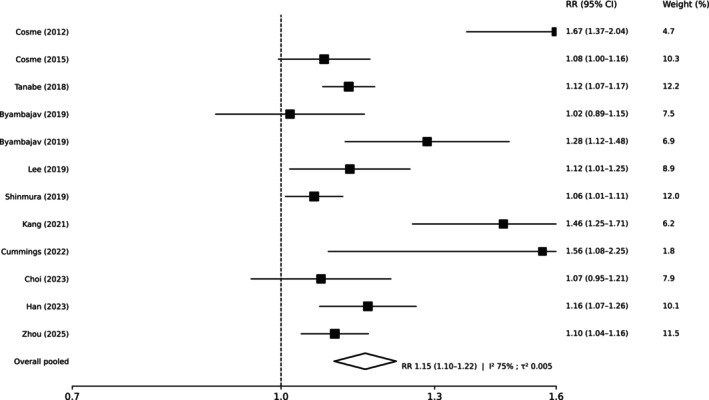
Forest plot of comparative non‐randomized studies assessing susceptibility‐guided versus empirical first‐line therapy, reporting intention‐to‐treat eradication. Study‐specific risk ratios (RR) with 95% confidence intervals (CI) are shown, with square size proportional to study weight; the diamond represents the pooled random‐effects estimate. In studies including multiple empirical regimen families, family‐specific contrasts are presented as separate lines. Results from non‐randomized studies are shown separately from randomized trials due to differences in risk of bias and causal inference.

In the primary ITT meta‐analysis, RCTs (k = 33) favored guided therapy over empirical regimens (pooled RR 1.09, 95% CI 1.05–1.13; I^2^ 73%; 95% PI 0.92–1.30; Figure 2). NRS (k = 12) showed a consistent direction with a larger magnitude (pooled RR 1.15, 95% CI 1.10–1.22; I^2^ 75%; 95% PI 0.99–1.35); (Figure 3). Because the outcome is eradication, RR > 1.00 favors guided therapy.

Pre‐specified subgroups in RCTs showed effect modification by comparator family. Against BQT, the average effect was neutral‐to‐modest (RR 1.03, 95% CI 0.97–1.10; I^2^ 67%), whereas against other empirical regimens it was more clearly favorable (RR 1.12, 95% CI 1.06–1.18; I^2^ 75%; Figure S1). By specimen, estimates were RR 1.08 (95% CI 1.04–1.13; I^2^ 74%) for biopsy (k ≈29), RR 1.27 (95% CI 1.06–1.51; I^2^ 54%) for gastric juice/string (k ≈2), and RR 1.16 (95% CI 0.94–1.41; I^2^ 67%) for stool (k ≈2). By method, effects were similar with culture/E‐test (RR 1.10, 95% CI 1.04–1.17; I^2^ 65%; k ≈16) and PCR (RR 1.09, 95% CI 1.03–1.14; I^2^ 78%; k ≈17). The observed heterogeneity (I^2^ 65%–78%) likely reflects differences in background resistance, empirical regimens, and susceptibility panels and matrices across settings.

Findings were consistent in sensitivity analyses. Restricting RCTs to BQT comparators yielded neutral‐to‐modest effects (consistent with a high‐performing empirical regimen) whereas restriction to non‐BQT comparators accentuated the benefit of guidance. Directionality was preserved when limiting by specimen/method (e.g., biopsy‐PCR only) and when excluding comparisons requiring continuity correction for zero cells. Exploratory assessments of small‐study effects (funnel plots and Egger's test where ≥ 10 studies were available) did not suggest major asymmetry in the primary RCT synthesis; Egger's regression test was not statistically significant (intercept 0.83, *p* = 0.30). The corresponding funnel plot is shown in Figure S2. Visual asymmetry in some subgroups was difficult to interpret given heterogeneity and limited study counts, and formal bias tests were not applied to strata with fewer than 10 studies. Where reported, PP analyses were directionally consistent with ITT (with magnitude varying by setting); inference was centered on ITT given the potential for attrition bias in PP populations.

Risk‐of‐bias assessments with RoB 2 for RCTs were generally low to some concerns (primarily deviations from intended interventions and outcome measurement), and ROBINS‐I for NRS indicated expected confounding and selection risks. Using GRADE, the certainty for the primary outcome was judged moderate for RCTs and low‐to‐moderate for NRS, acknowledging the heterogeneity and potential residual confounding. Risk of bias assessments are summarized in Table S2; overall certainty judgments are provided in Table S1.

## 4 Discussion

In this adult first‐line setting, susceptibility‐guided therapy achieved equal or superior eradication compared with empirical treatment. The pooled effect across RCTs was modest but consistent (RR ≈1.09). For illustrative purposes, applying this relative effect to a baseline eradication rate of approximately 80%—a value commonly reported for empirical regimens in contemporary studies—would correspond to an absolute increase of around 7–8 percentage points and a number needed to treat of approximately 13. Absolute effects will vary according to baseline eradication rates in different settings. Estimates from non‐randomized studies were larger in magnitude yet directionally concordant, which supports a genuine treatment advantage while reinforcing that inference should rely primarily on randomized evidence. These results agree with earlier syntheses that suggested benefit for tailored therapy overall, particularly when empirical regimens are not optimized [57].

The comparator influences the observed effect. When the comparator was BQT, effects were neutral to modest, which is consistent with a high‐performing empirical regimen that is relatively resilient to resistance. Against non‐BQT comparators, the advantage of guidance was clearer. This gradient mirrors the Nyssen et al. meta‐analysis, which found that susceptibility‐guided strategies were generally more effective than empirical therapy overall, but not clearly superior to contemporary first‐line quadruple regimens once older triple therapy was excluded, a pattern also seen within randomized trials [58]. A focused 2022 analysis comparing guided therapy specifically with BQT reached similar conclusions, with pooled effects close to unity when genotypic panels were limited to 23S detection, and stronger signals where culture or E‐test informed broader tailoring across multiple antibiotics. This is consistent with the idea that tailoring adds value, but the content of the test and how it informs regimen choice influence the size of the effect [59]. In clinical practice, resistance testing targeting key antibiotics with high resistance rates—particularly clarithromycin and fluoroquinolones—may capture most of the clinically relevant variability in treatment response, and several included studies focused primarily on these determinants, supporting the concept that targeted testing may be sufficient in many settings. Our specimen and method subgroups, including biopsy, stool, and gastric juice, as well as culture/E‐test and PCR approaches, were directionally similar. However, analyses based on stool and gastric juice specimens were informed by a small number of studies and should therefore be interpreted with caution, as the available evidence remains limited. The reliability of non‐invasive susceptibility testing using stool or gastric juice remains an area of ongoing investigation. While molecular approaches have shown promising concordance with biopsy‐based testing in some studies, variability in assay performance and sample quality may influence accuracy, and further validation is required before routine clinical implementation.

The non‐bismuth comparator group comprised heterogeneous regimens, including clarithromycin‐based triple therapy, concomitant therapy, and potassium‐competitive acid blocker–based regimens, which differ substantially in efficacy and resistance vulnerability. Although further stratification would be clinically informative, inconsistent reporting and limited sample sizes within individual regimen categories precluded robust subgroup analyses. This heterogeneity likely contributes to the larger observed effect size in the non‐BQT subgroup and should be considered when interpreting these results.

Heterogeneity was substantial (I^2^ around 65%–78%), as expected given differences in resistance ecology, empirical regimens, and susceptibility‐testing strategies across regions. Additional sources of variability likely include geographic differences in antimicrobial resistance patterns, variation in treatment duration and acid suppression, and differences between phenotypic and genotypic susceptibility testing approaches. The heterogeneity within the non‐bismuth comparator group, which includes regimens with markedly different baseline efficacy, may also have contributed to the observed variability. Reporting prediction intervals shows that, while the average effect favors guidance, the true effect in a new setting may range from minimal to clinically important benefit. This interpretation is aligned with the Maastricht VI/Florence guideline: when individual susceptibility is unavailable, BQT is recommended as first‐line therapy in settings with high or unknown clarithromycin resistance; routine susceptibility testing is reasonable and aligned with stewardship goals, but its widespread use depends on local performance of empirical regimens and logistics [4].

Sensitivity analyses supported the robustness of our findings. Restricting RCTs to BQT comparators produced neutral to modest effects, which is consistent with the high performance of BQT, whereas restricting to non‐BQT comparators accentuated the benefit of guidance. Limiting by specimen or method preserved directionality, and excluding comparisons that required continuity correction did not materially change results. Exploratory checks for small‐study effects did not suggest major asymmetry in the primary randomized synthesis; where visual asymmetry appeared in subgroups, interpretation was limited by heterogeneity and small numbers. Where reported, per‐protocol estimates aligned in direction with ITT, and inference remained centered on ITT to avoid attrition bias.

This review has several strengths, including a comprehensive search across three databases without language restriction, duplicate screening with adjudication, explicit handling of multi‐arm trials to avoid unit‐of‐analysis errors, prioritization of ITT outcomes, separate syntheses for randomized and non‐randomized evidence, prespecified subgrouping by comparator and testing domain, and the use of prediction intervals alongside pooled effects. Limitations include residual heterogeneity despite subgrouping, reliance on non‐randomized evidence in some strata, sparse data for stool and gastric juice matrices, and incomplete reporting of adherence, adverse events, and costs. These constraints are common to the field and help explain differences across settings and across prior reviews.

From a health systems perspective, susceptibility‐guided therapy entails additional costs and logistical requirements, including endoscopy or molecular testing. Its cost‐effectiveness is therefore likely context‐dependent, being more favorable in settings with high resistance prevalence or lower empirical eradication rates, and less advantageous where highly effective empirical regimens such as bismuth quadruple therapy are widely available.

Clinically, the data support context‐specific adoption. Systems already achieving high eradication with BQT can prioritize targeted use of susceptibility testing, for example in patients with prior macrolide exposure, in penicillin allergy that limits options, or in regions with high fluoroquinolone resistance. Settings that rely on non‐BQT regimens, or that face high resistance to key agents, are more likely to realize meaningful gains from broader susceptibility guidance. This interpretation is consistent with both the Nyssen et al. review and Maastricht VI/Florence guideline. The 2021 randomized evidence synthesis similarly suggested slight overall superiority for guided therapy and superiority over clarithromycin‐based triple regimens in high‐resistance contexts, but no superiority over quadruple therapy, which is reproduced by our results [57]. The 2022 comparison of guided therapy with BQT also found estimates close to unity overall, with larger benefits when culture or E‐test enabled tailoring beyond macrolides alone [59].

In conclusion, for adult first‐line eradication of *Helicobacter pylori* , susceptibility‐guided therapy is at least non‐inferior and often superior to empirical therapy. The incremental benefit is smallest against BQT and largest where non‐BQT regimens predominate or resistance to key agents is high. Implementation should be determined locally, balancing resistance epidemiology, the empirical regimen in use, the scope and logistics of testing, and antimicrobial‐stewardship priorities, which is consistent with and extends prior evidence syntheses and contemporary consensus recommendations. Supporting Information is available online and includes the full search strategies (Appendix S1), detailed methodological specifications (Appendix S2), the GRADE summary of findings (Table S1), risk of bias assessments (Table S2), and additional figures including a forest plot of randomized controlled trials stratified by empirical regimen family (Figure S1) and a funnel plot of randomized controlled trials (Figure S2).

## Author Contributions

Manuel Coelho Rocha and Tiago Gaspar contributed to study conception and design, literature screening, data extraction, and data analysis. Carlos Bernardes acted as third reviewer for conflict resolution during study selection. Manuel Coelho Rocha drafted the manuscript, and all authors contributed to the interpretation of data, critically revised the manuscript for important intellectual content, and approved the final version for submission.

## Supporting information


**Appendix S1:** hel70125‐sup‐0001‐AppendixS1.docx.

**Appendix S2:** hel70125‐sup‐0002‐AppendixS2.docx.

**FIGURE S1:** Forest plot of randomized controlled trials stratified by empirical regimen family (bismuth quadruple therapy [BQT] vs. non‐BQT regimens). Within each stratum, study‐specific risk ratios (RR) with 95% confidence intervals (CI) and pooled random‐effects estimates are displayed. Trials including both empirical families contribute separate family‐specific contrasts, consistent with the prespecified multi‐arm handling approach. Heterogeneity statistics (I^2^ and τ^2^) are shown for each subgroup.

**FIGURE S2:** Funnel plot of randomized controlled trials assessing susceptibility‐guided versus empirical first‐line therapy. Study‐specific effect sizes (log risk ratio) are plotted against their standard errors. The dashed vertical line represents the pooled effect estimate, and diagonal lines indicate pseudo 95% confidence limits. Visual inspection did not suggest major asymmetry, consistent with Egger's regression test (*p* = 0.30).

**TABLE S1:** GRADE summary of findings. Certainty of evidence for the primary outcome (intention‐to‐treat eradication) was assessed using the GRADE approach. Because baseline eradication rates varied substantially across settings and comparator regimens, absolute effects were not presented in this summary table; pooled relative effects are therefore reported as the primary measure of effect.

**TABLE S2:** Risk of bias assessment. Risk of bias was assessed using the Cochrane RoB 2 tool for randomized controlled trials and the ROBINS‐I tool for non‐randomized comparative studies.

## Data Availability

The extracted study‐level dataset and statistical code supporting the findings of this study are available from the corresponding author upon reasonable request.
