# Supplementary material for: Susceptibility‐Guided Versus Empirical First‐Line Therapy of Helicobacter pylori Infection in Adults: A Systematic Review and Meta‐Analysis
Source: Helicobacter. 2026 Apr 14;31(2):e70125. doi: 10.1111/hel.70125 (PMC13080058; doi:10.1111/hel.70125)
Supplement: Supplementary file 1 — Appendix S1: hel70125‐sup‐0001‐AppendixS1.docx. [file HEL-31-e70125-s001.docx]

**Supplementary Appendix 1**

**Full search strategies (last search: 30 September 2025)**

Searches were conducted in MEDLINE (via PubMed), Web of Science Core Collection, and Scopus from database inception to 30 September 2025. No language restrictions were applied. Searches combined controlled vocabulary (where applicable) and structured title/abstract terms for Helicobacter pylori, antimicrobial resistance/susceptibility testing, and susceptibility-guided versus empirical therapy. Human filters were applied where available.

**MEDLINE (PubMed)**

The following structured strategy was used:

("Helicobacter pylori"[MeSH Terms] OR "Helicobacter pylori"[Title/Abstract] OR "H pylori"[Title/Abstract] OR "Campylobacter pylori"[Title/Abstract])
AND
("drug resistance, bacterial"[MeSH Terms] OR resistance[Title/Abstract] OR susceptib*[Title/Abstract] OR "antimicrobial susceptibility"[Title/Abstract] OR "antibiotic susceptibility"[Title/Abstract] OR antibiogram*[Title/Abstract] OR culture[Title/Abstract] OR "E-test"[Title/Abstract] OR "agar dilution"[Title/Abstract] OR MIC[Title/Abstract] OR PCR[Title/Abstract] OR "polymerase chain reaction"[Title/Abstract] OR 23S[Title/Abstract] OR gyrA[Title/Abstract] OR rdxA[Title/Abstract] OR pbp1A[Title/Abstract] OR 16S[Title/Abstract] OR rpoB[Title/Abstract])
AND
(guided[Title/Abstract] OR tailored[Title/Abstract] OR "susceptibility-guided"[Title/Abstract] OR "resistance-guided"[Title/Abstract] OR "genotype-guided"[Title/Abstract] OR empirical[Title/Abstract] OR "standard therapy"[Title/Abstract])

Animal-only studies were excluded using the filter:
NOT (animals[mh] NOT humans[mh])

Structured runs targeting randomized controlled trials were also performed using standard RCT filters and combined with the main search set.

________________________________________________________________

**Scopus**

Searches were conducted using TITLE-ABS-KEY fields combining:

- “Helicobacter pylori”
- resistance OR susceptibility OR PCR OR culture OR E-test OR MIC OR 23S OR gyrA OR rdxA OR pbp1A
- guided OR tailored OR genotype-guided OR susceptibility-guided OR empirical OR standard therapy

Separate structured runs were performed to ensure comprehensive capture of comparative first-line studies and were combined prior to deduplication.

________________________________________________________________

**Web of Science Core Collection**

Searches were performed using the Topic field (TS=) combining:

- TS=("Helicobacter pylori")
  AND
- TS=(resistance OR susceptibility OR PCR OR culture OR MIC OR 23S OR gyrA OR rdxA OR pbp1A)
  AND
- TS=(guided OR tailored OR empirical OR "standard therapy")

Multiple structured runs were performed to capture randomized and comparative non-randomized studies and were combined before export.

All retrieved records from each database were exported and merged. Deduplication was performed prior to screening.
