# Supplementary material for: Susceptibility‐Guided Versus Empirical First‐Line Therapy of Helicobacter pylori Infection in Adults: A Systematic Review and Meta‐Analysis
Source: Helicobacter. 2026 Apr 14;31(2):e70125. doi: 10.1111/hel.70125 (PMC13080058; doi:10.1111/hel.70125)
Supplement: Supplementary file 2 — Appendix S2: hel70125‐sup‐0002‐AppendixS2.docx. [file HEL-31-e70125-s005.docx]

**Supplementary Appendix 2**

**Additional methodological details**

Multi-arm trials were handled to avoid unit-of-analysis errors. When a study included multiple empirical regimens within the same therapeutic family, empirical arms were combined into a single comparator group (summing totals and eradication counts) so that the susceptibility-guided arm was not double-counted. When distinct empirical families co-existed within the same study (e.g., bismuth quadruple therapy [BQT] and non-BQT regimens), each family-specific contrast was entered as a separate comparison following a prespecified hierarchy, and each comparison contributed once to the relevant subgroup synthesis.

For comparisons with a zero cell in the 2×2 table, a continuity correction of 0.5 was applied to all four cells prior to calculating the log risk ratio and its standard error, as prespecified. Sensitivity analyses excluded comparisons requiring continuity correction to evaluate robustness.

Intention-to-treat (ITT) eradication was prioritized as the primary outcome to preserve randomization (where applicable) and minimize attrition bias. Per-protocol (PP) eradication was extracted when reported and summarized descriptively but was not pooled as the primary evidence base.

The effect measure was the risk ratio (RR) for eradication. Study-specific log(RR) values were pooled using a DerSimonian–Laird random-effects model. Between-study heterogeneity was quantified using I² and τ². Where applicable, 95% prediction intervals were calculated to reflect the expected range of effects in similar future settings.

Randomized controlled trials (RCTs) and comparative non-randomized studies (NRS) were analyzed separately, reflecting differences in risk of bias and causal inference. The primary meta-analysis was based on RCTs; NRS were synthesized as supportive evidence.

Genotypic susceptibility testing targeted mutations associated with resistance to clarithromycin (23S rRNA point mutations including A2142G, A2142C, and A2143G) and fluoroquinolones (gyrA mutations), with additional resistance determinants evaluated in some studies, including metronidazole (rdxA and frxA), amoxicillin (pbp1A), tetracycline (16S rRNA), and rifabutin (rpoB), according to the testing panels employed by each study.

Prespecified subgroup analyses examined empirical comparator family (BQT vs non-BQT) and the susceptibility testing domain (specimen and method). Sensitivity analyses restricted the synthesis to BQT-only comparators, to biopsy-based PCR-only guidance, and excluded comparisons requiring continuity correction.

Screening was conducted in Rayyan by two reviewers with third-reviewer adjudication. Meta-analyses were performed in R using established meta-analysis packages.

All analytical decisions described above (including outcome prioritization, multi-arm handling, subgroup definitions, and sensitivity analyses) were prespecified in the registered protocol (PROSPERO CRD420251138257) and were implemented as planned.
