# Supplementary material for: Susceptibility‐Guided Versus Empirical First‐Line Therapy of Helicobacter pylori Infection in Adults: A Systematic Review and Meta‐Analysis
Source: Helicobacter. 2026 Apr 14;31(2):e70125. doi: 10.1111/hel.70125 (PMC13080058; doi:10.1111/hel.70125)

**Supplementary Figure 1** - Forest plot of randomized controlled trials stratified by empirical regimen family (bismuth quadruple therapy [BQT] vs non-BQT regimens). Within each stratum, study-specific risk ratios (RR) with 95% confidence intervals (CI) and pooled random-effects estimates are displayed. Trials including both empirical families contribute separate family-specific contrasts, consistent with the prespecified multi-arm handling approach. Heterogeneity statistics (I² and τ²) are shown for each subgroup.


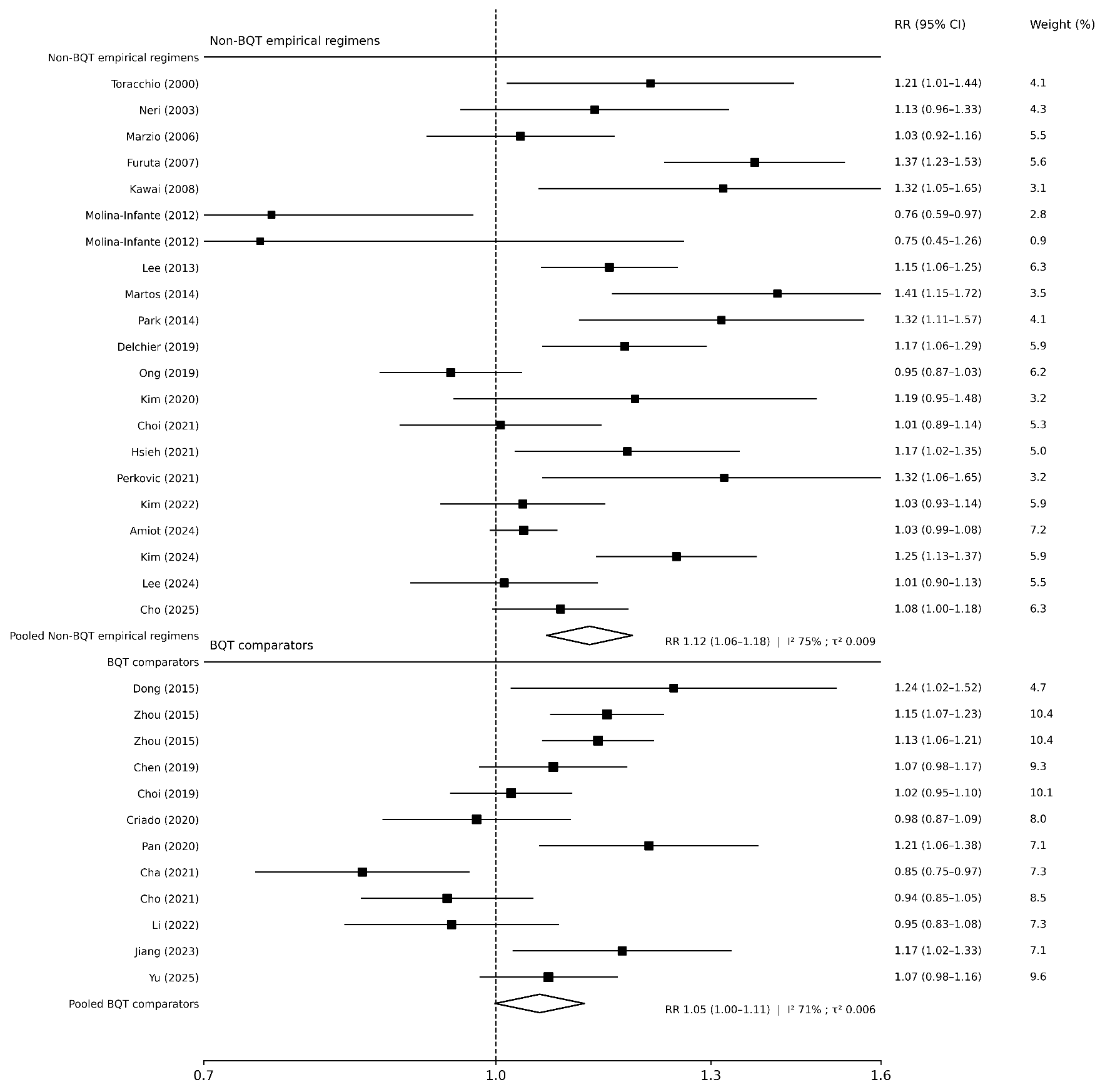

Supplement: Supplementary file 3 — FIGURE S1: Forest plot of randomized controlled trials stratified by empirical regimen family (bismuth quadruple therapy [BQT] vs. non‐BQT regimens). Within each stratum, study‐specific risk ratios (RR) with 95% confidence intervals (CI) and pooled random‐effects estimates are displayed. Trials including both empirical families contribute separate family‐specific contrasts, consistent with the prespecified multi‐arm handling approach. Heterogeneity statistics (I2 and τ2) are shown for each subgroup. [file HEL-31-e70125-s003.docx]
