# Supplementary material for: Susceptibility‐Guided Versus Empirical First‐Line Therapy of Helicobacter pylori Infection in Adults: A Systematic Review and Meta‐Analysis
Source: Helicobacter. 2026 Apr 14;31(2):e70125. doi: 10.1111/hel.70125 (PMC13080058; doi:10.1111/hel.70125)

**Supplementary Figure 2** - Funnel plot of randomized controlled trials assessing susceptibility-guided versus empirical first-line therapy. Study-specific effect sizes (log risk ratio) are plotted against their standard errors. The dashed vertical line represents the pooled effect estimate, and diagonal lines indicate pseudo 95% confidence limits. Visual inspection did not suggest major asymmetry, consistent with Egger’s regression test (p = 0.30).


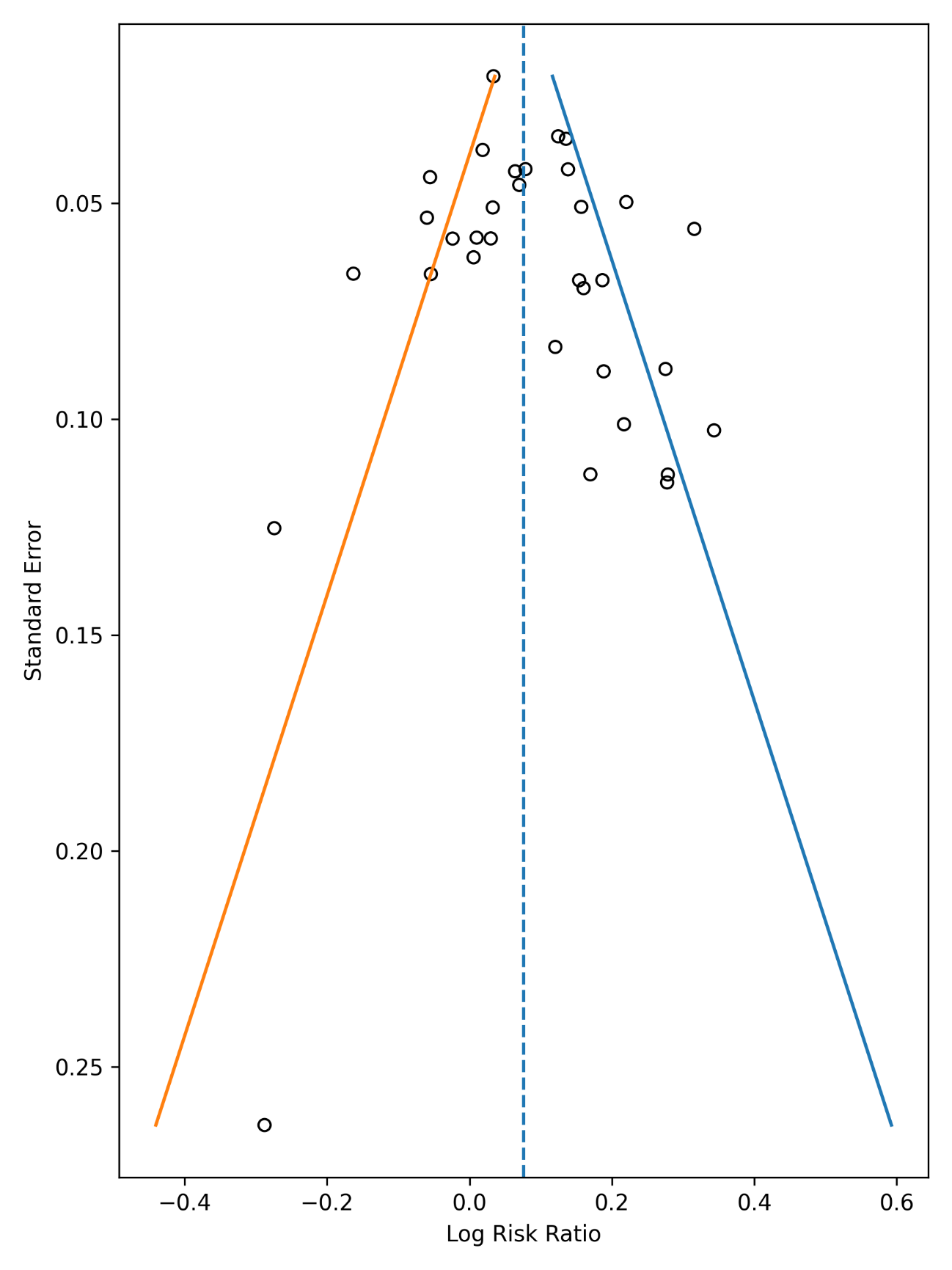

Supplement: Supplementary file 4 — FIGURE S2: Funnel plot of randomized controlled trials assessing susceptibility‐guided versus empirical first‐line therapy. Study‐specific effect sizes (log risk ratio) are plotted against their standard errors. The dashed vertical line represents the pooled effect estimate, and diagonal lines indicate pseudo 95% confidence limits. Visual inspection did not suggest major asymmetry, consistent with Egger's regression test (p = 0.30). [file HEL-31-e70125-s004.docx]
