# Supplementary material for: Susceptibility‐Guided Versus Empirical First‐Line Therapy of Helicobacter pylori Infection in Adults: A Systematic Review and Meta‐Analysis
Source: Helicobacter. 2026 Apr 14;31(2):e70125. doi: 10.1111/hel.70125 (PMC13080058; doi:10.1111/hel.70125)
Supplement: Supplementary file 5 — TABLE S1: GRADE summary of findings. Certainty of evidence for the primary outcome (intention‐to‐treat eradication) was assessed using the GRADE approach. Because baseline eradication rates varied substantially across settings and comparator regimens, absolute effects were not presented in this summary table; pooled relative effects are therefore reported as the primary measure of effect. [file HEL-31-e70125-s006.docx]

**Supplementary Table 1 - GRADE summary of findings.** Certainty of evidence for the primary outcome (intention-to-treat eradication) was assessed using the GRADE approach. Because baseline eradication rates varied substantially across settings and comparator regimens, absolute effects were not presented in this summary table; pooled relative effects are therefore reported as the primary measure of effect.

| **Comparison** | **Study design** | **No. of studies** | **Relative effect (RR, 95% CI)** | **Absolute effect** | **Certainty (GRADE)** | **Reasons for downgrading** |
| --- | --- | --- | --- | --- | --- | --- |
| Susceptibility-guided vs empirical (overall) | Randomized trials (RCTs) | 33 | RR 1.09 (1.05–1.13) | Not computed (baseline varies) | Moderate | Downgraded 1 level for inconsistency (substantial heterogeneity). |
| Susceptibility-guided vs BQT empirical | Randomized trials (RCTs) | 10 | RR 1.03 (0.97–1.10) | Not computed (baseline varies) | Moderate | Downgraded for inconsistency; effect small and CI compatible with minimal benefit. |
| Susceptibility-guided vs non‑BQT empirical | Randomized trials (RCTs) | 21 | RR 1.12 (1.06–1.18) | Not computed (baseline varies) | Moderate | Downgraded 1 level for inconsistency (heterogeneity). |
| Susceptibility-guided vs empirical (overall) | Non-randomized studies (NRS) | 12 | RR 1.15 (1.10–1.22) | Not computed (baseline varies) | Low | Downgraded for serious risk of bias (confounding) and inconsistency. |
