# Supplementary material for: Susceptibility‐Guided Versus Empirical First‐Line Therapy of Helicobacter pylori Infection in Adults: A Systematic Review and Meta‐Analysis
Source: Helicobacter. 2026 Apr 14;31(2):e70125. doi: 10.1111/hel.70125 (PMC13080058; doi:10.1111/hel.70125)
Supplement: Supplementary file 6 — TABLE S2: Risk of bias assessment. Risk of bias was assessed using the Cochrane RoB 2 tool for randomized controlled trials and the ROBINS‐I tool for non‐randomized comparative studies. [file HEL-31-e70125-s002.docx]

**Supplementary Table 2 - Risk of bias assessment.** Risk of bias was assessed using the Cochrane RoB 2 tool for randomized controlled trials and the ROBINS-I tool for non-randomized comparative studies.

Randomized controlled trials (RoB 2):

| **Study** | **Randomization process** | **Deviations from intended interventions** | **Missing outcome data** | **Measurement of the outcome** | **Selection of the reported result** | **Overall RoB 2** |
| --- | --- | --- | --- | --- | --- | --- |
| Amiot (2024) | Low | Some concerns | Low | Some concerns | Low | Some concerns |
| Cha (2021) | Low | Some concerns | Low | Some concerns | Low | Some concerns |
| Chen (2019) | Low | Some concerns | Low | Some concerns | Low | Some concerns |
| Cho (2021) | Low | Some concerns | Low | Some concerns | Low | Some concerns |
| Cho (2025) | Low | Some concerns | Low | Some concerns | Low | Some concerns |
| Choi (2019) | Low | Some concerns | Low | Some concerns | Low | Some concerns |
| Choi (2021) | Low | Some concerns | Low | Some concerns | Low | Some concerns |
| Criado (2020) | Low | Some concerns | Low | Some concerns | Low | Some concerns |
| Delchier (2019) | Low | Some concerns | Low | Some concerns | Low | Some concerns |
| Dong (2015) | Low | Some concerns | Low | Some concerns | Low | Some concerns |
| Furuta (2007) | Low | Some concerns | Low | Some concerns | Low | Some concerns |
| Hsieh (2021) | Low | Some concerns | Low | Some concerns | Low | Some concerns |
| Jiang (2023) | Low | Some concerns | Low | Some concerns | Low | Some concerns |
| Kawai (2008) | Low | Some concerns | Low | Some concerns | Low | Some concerns |
| Kim (2020) | Low | Some concerns | Low | Some concerns | Low | Some concerns |
| Kim (2022) | Low | Some concerns | Low | Some concerns | Low | Some concerns |
| Kim (2024) | Low | Some concerns | Low | Some concerns | Low | Some concerns |
| Lee (2013) | Low | Some concerns | Low | Some concerns | Low | Some concerns |
| Lee (2024) | Low | Some concerns | Low | Some concerns | Low | Some concerns |
| Li (2022) | Low | Some concerns | Low | Some concerns | Low | Some concerns |
| Martos (2014) | Low | Some concerns | Low | Some concerns | Low | Some concerns |
| Marzio (2006) | Low | Some concerns | Low | Some concerns | Low | Some concerns |
| Molina-Infante (2012) | Low | Some concerns | Low | Some concerns | Low | Some concerns |
| Neri (2003) | Low | Some concerns | Low | Some concerns | Low | Some concerns |
| Ong (2019) | Low | Some concerns | Low | Some concerns | Low | Some concerns |
| Pan (2020) | Low | Some concerns | Low | Some concerns | Low | Some concerns |
| Park (2014) | Low | Some concerns | Low | Some concerns | Low | Some concerns |
| Perkovic (2021) | Low | Some concerns | Low | Some concerns | Low | Some concerns |
| Toracchio (2000) | Low | Some concerns | Low | Some concerns | Low | Some concerns |
| Yu (2025) | Low | Some concerns | Low | Some concerns | Low | Some concerns |
| Zhou (2015) | Low | Some concerns | Low | Some concerns | Low | Some concerns |

| **Study** | **Confounding** | **Selection of participants** | **Classification of interventions** | **Deviations from intended interventions** | **Missing data** | **Measurement of outcomes** | **Overall ROBINS‑I** |
| --- | --- | --- | --- | --- | --- | --- | --- |
| Byambajav (2019) | Serious | Moderate | Low | Moderate | Low | Moderate | Serious |
| Choi (2023) | Serious | Moderate | Low | Moderate | Low | Moderate | Serious |
| Cosme (2012) | Serious | Moderate | Low | Moderate | Low | Moderate | Serious |
| Cosme (2015) | Serious | Moderate | Low | Moderate | Low | Moderate | Serious |
| Cummings (2022) | Serious | Moderate | Low | Moderate | Low | Moderate | Serious |
| Han (2023) | Serious | Moderate | Low | Moderate | Low | Moderate | Serious |
| Kang (2021) | Serious | Moderate | Low | Moderate | Low | Moderate | Serious |
| Lee (2019) | Serious | Moderate | Low | Moderate | Low | Moderate | Serious |
| Shinmura (2019) | Serious | Moderate | Low | Moderate | Low | Moderate | Serious |
| Tanabe (2018) | Serious | Moderate | Low | Moderate | Low | Moderate | Serious |
| Zhou (2025) | Serious | Moderate | Low | Moderate | Low | Moderate | Serious |

Note: Several studies shared identical risk-of-bias judgments because they had comparable designs, outcome ascertainment methods, and reporting standards. Assessments were performed at the study level following RoB 2 and ROBINS-I guidance.

Non-randomized comparative studies (ROBINS‑I):
